# Single-Center Pilot Evaluation of Virtual Vascular Surgery Rotation in Retention of Knowledge and Skills

**DOI:** 10.7759/cureus.112073

**Published:** 2026-07-05

**Authors:** Joel Harding, Amrita K Mahajan, Christopher Noty, Dongjin Suh, Alexander Neifert, John Cabot, Misty Humphries

**Affiliations:** 1 Department of Vascular Surgery, University of California, Davis, Davis, USA; 2 Department of Surgery, Henry Ford Health System, Wyandotte, USA; 3 Department of General Surgery, Penn State Health Milton S. Hershey Medical Center, Hershey, USA; 4 Department of Surgery, University of Iowa Hospitals & Clinics, Iowa City, USA; 5 Department of Surgery, Yale School of Medicine, New Haven, USA; 6 Department of Surgery, Stanford University Medical Center, Palo Alto, USA

**Keywords:** assessment in health professions education, general and vascular surgery, instructional methods in health professions education, surgical-education, virtual rotation

## Abstract

Background: The COVID-19 pandemic substantially disrupted surgical education, primarily by limiting in-person rotations, prompting the development of virtual learning platforms. The University of California Davis Vascular Surgery Department piloted a two-week virtual vascular surgery rotation to evaluate its effectiveness in improving surgical skills and knowledge retention among fourth-year medical students nationwide.

Methods: Five medical students from across the United States enrolled and participated in the virtual rotation. Baseline assessments were obtained, including a 37-question multiple-choice examination covering core vascular topics and timed evaluations of one-handed and two-handed knot-tying skills. The virtual curriculum included interactive sessions, such as case conferences, virtual rounding, faculty- and resident-led didactics, and journal clubs, as well as non-interactive components (readings, recorded surgical videos, and written assignments). Students were reassessed post-rotation and at six months. Statistical analysis was performed using mean comparisons and Fisher's exact testing, with significance set at p<0.05.

Results: Students demonstrated statistically significant improvements in both one-handed and two-handed knot-tying skills at the two-week and six-month time points compared to baseline (p<0.05). Knowledge scores improved at two weeks (p=0.012) but declined by six months; there was no significant difference from baseline (p=0.32). All students remained above baseline in both skills and knowledge at six months despite measurable regression from the two-week assessment. Interactive sessions were rated more favorably than non-interactive activities (p=0.003). All participants reported that the rotation met expectations.

Conclusions: Virtual education has become an integral part of modern education, including the surgical realm. A virtual surgical rotation can produce sustained progress and improvement in surgical skills and knowledge base. While knowledge retention may decline over time with the virtual format alone, this only reinforces the need for multimodal education elements and long-term learning that may incorporate reading and continued clinical/surgical exposure. Interactive components appear to be critical to the perceived educational value of virtual surgical rotations as well.

## Introduction

The 2020 year of surgical education was hampered by the novel SARS-CoV-2 (COVID-19) pandemic. Medical students were unable to rotate in person. Conferences were held virtually, while pre-clerkship and clerkship education were replaced by online modules [1,2]. New challenges created new solutions, and virtual rotations were put in the spotlight for fourth-year medical students eager to exhibit their interests in surgical specialties. Virtual rotations are not a new idea, but their utilization was limited to imaging and didactic-based learning [3,4]. Surgical education craves in-person and hands-on learning experiences, so novel techniques were developed to accommodate this, including live-streamed operating room cases, virtual rounds with surgical teams, telemedicine visits, and live virtual tutorials on hand skills [5,6].

The University of California Davis Vascular Surgery Department implemented a virtual vascular surgery rotation for the 2020-2021 academic year. This rotation combined hands-on training in surgical skills, exposure to vascular surgery through faculty-led didactic lectures, surgical videos, imaging review, virtual patient rounding, and virtual patient interactions. Five students from across the United States participated. Pre-rotation, immediate post-rotation, and six-month follow-up assessments were performed to evaluate outcomes.

The objectives of this exploratory pilot study are (1) to assess short-term and six-month retention of vascular surgery knowledge, (2) to evaluate retention of knot-tying skills, and (3) to characterize learner perceptions of the virtual rotation format and its educational activities. These aims are intended to generate hypotheses and inform future research, rather than establish efficacy.

This article was previously presented as a meeting abstract at the 2022 Vascular Annual Meeting on June 16, 2022.

## Materials and methods

Five students participated in the program from various regions in the United States: the West Coast, Southeast, Midwest, and East Coast. They were recruited through either virtual residency fairs or networking among faculty at the involved institutions. All students were given a 37-question multiple-choice test covering the following categories: vascular anatomy, carotid disease, aortic disease, peripheral arterial disease, vascular trauma, and imaging. Next, students were given one-on-one sessions to assess their baseline knot-tying skills by completing as many one-handed and two-handed knots as possible in one minute. This was conducted through individual 10-minute sessions with one of the senior residents on the service, who would proctor the session. Using a shoelace or other thread, fabric, or suture material, the learner would have one minute to tie as many one-handed or two-handed ties as possible and then report the number.

The students then participated in a two-week virtual rotation consisting of interactive sessions, including case conferences, virtual rounding, faculty- and resident-led didactics, and journal clubs, as well as non-interactive sessions, including readings, watching pre-recorded surgical videos, and written assignments. Upon completion, they were re-assessed on their knowledge and skills, along with a six-month follow-up. Upon completion, a survey was administered to ask students which activities they considered effective or ineffective for their learning. The activities were separated into two groups: interactive and non-interactive. Fischer's exact test was used to determine statistical significance among the categories.

Data were analyzed in Excel (Microsoft® Corp., Redmond, WA) to calculate average scores and standard deviations for each follow-up category. Standard deviation, mean, and group size were used to calculate statistical significance between the baseline and follow-up groups. A p-value <0.05 was set to be statistically significant.

## Results

The group showed statistically significant improvement from baseline at two weeks and six-month follow-up in both one-handed (p=0.012/0.02) and two-handed (p=0.015/0.01) ties. Students, on average, showed a statistically significant improvement in knowledge from baseline to the two-week follow-up (p=0.012); however, at the six-month follow-up, there was no statistical change from baseline (p=0.32) (Table 1).

**Table 1:** Mean and standard deviation of the two-week and six-month follow-up groups compared with baseline

Group	Mean	Standard Deviation	p-value
Baseline			
Knowledge	27.6	2.88	
One-hand tie	19.4	6.34	
Two-hand tie	15.2	3.63	
2-Week Follow-Up			
Knowledge	33	2.23	0.012
One-hand tie	31.6	5.55	0.012
Two-hand tie	26	6.92	0.015
6-Month Follow-Up			
Knowledge	29.4	2.41	0.32
One-hand tie	30.8	6.30	0.02
Two-hand tie	26.8	6.80	0.01

Two-week follow-up compared to six-month follow-up showed no statistically significant improvements in the skills groups: one-hand tie (p=0.84) and two-hand tie (p=0.86). There was a statistically significant regression in knowledge from the two-week follow-up to the six-month follow-up (p=0.04) (Table 2).

**Table 2:** Mean and standard deviation of the two-week versus six-month follow-up groups

Group	Mean	Standard Deviation	p-value
2-Week Follow-Up			
Knowledge	33	2.23	
One-hand tie	31.6	5.55	
Two-hand tie	26	6.92	
6-Month Follow-Up			
Knowledge	29.4	2.41	0.04
One-hand tie	30.8	6.30	0.84
Two-hand tie	26.8	6.80	0.86

**Figure 1:**
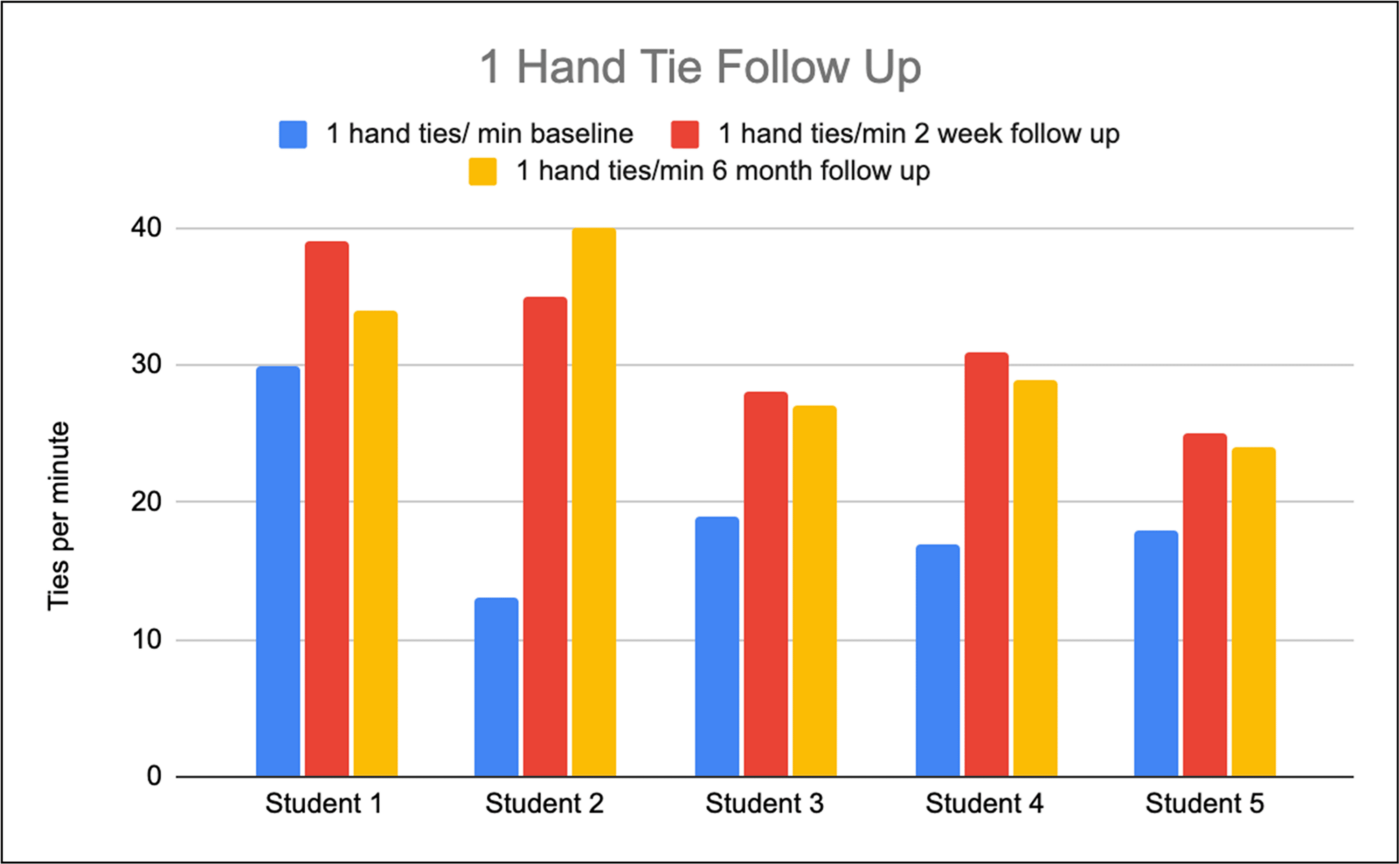
Follow-up of one-hand tie performance from baseline to six months

**Figure 2:**
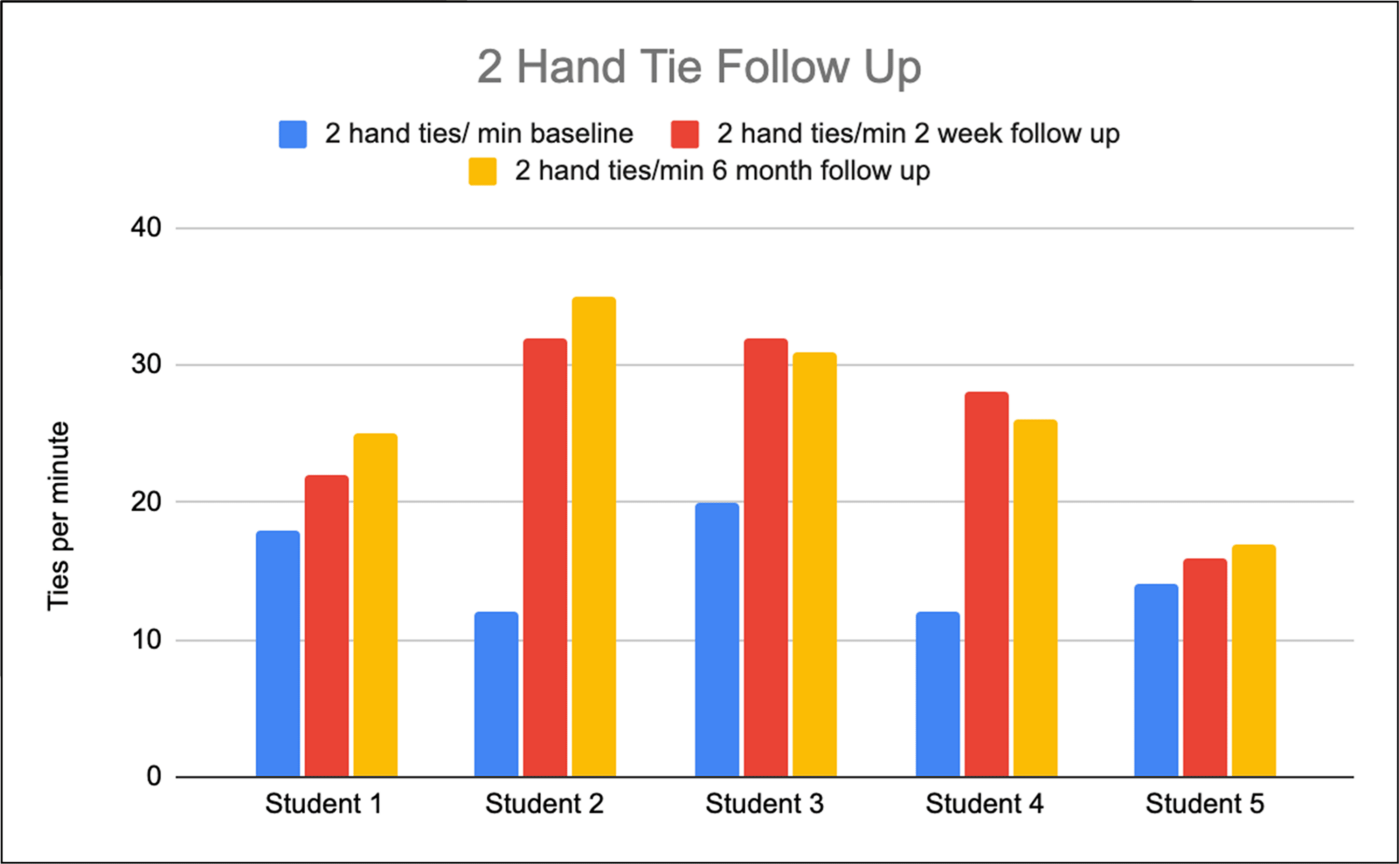
Follow-up of two-hand tie performance from baseline to six months

**Figure 3:**
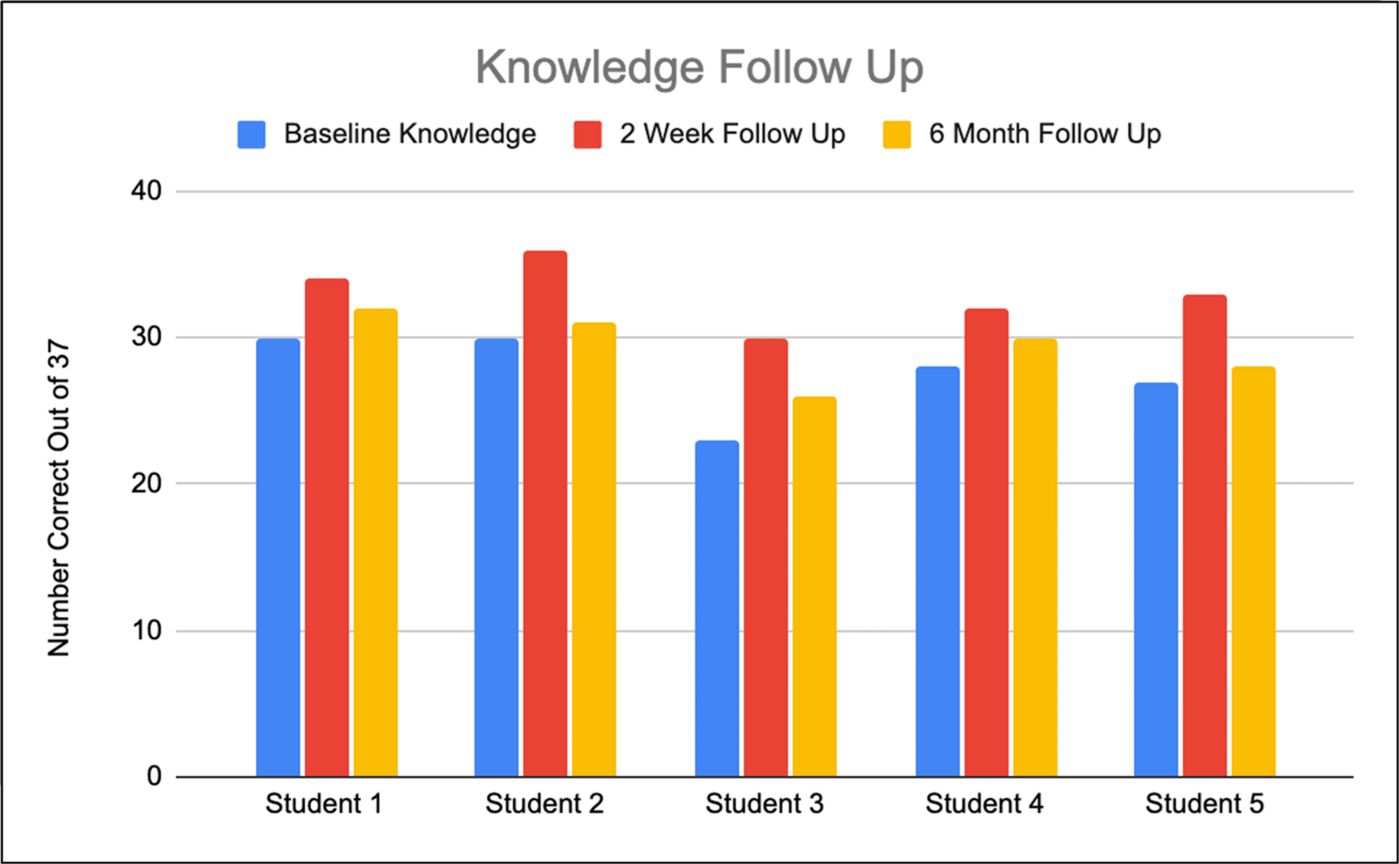
Follow-up of knowledge from baseline to six months

**Figure 4:**
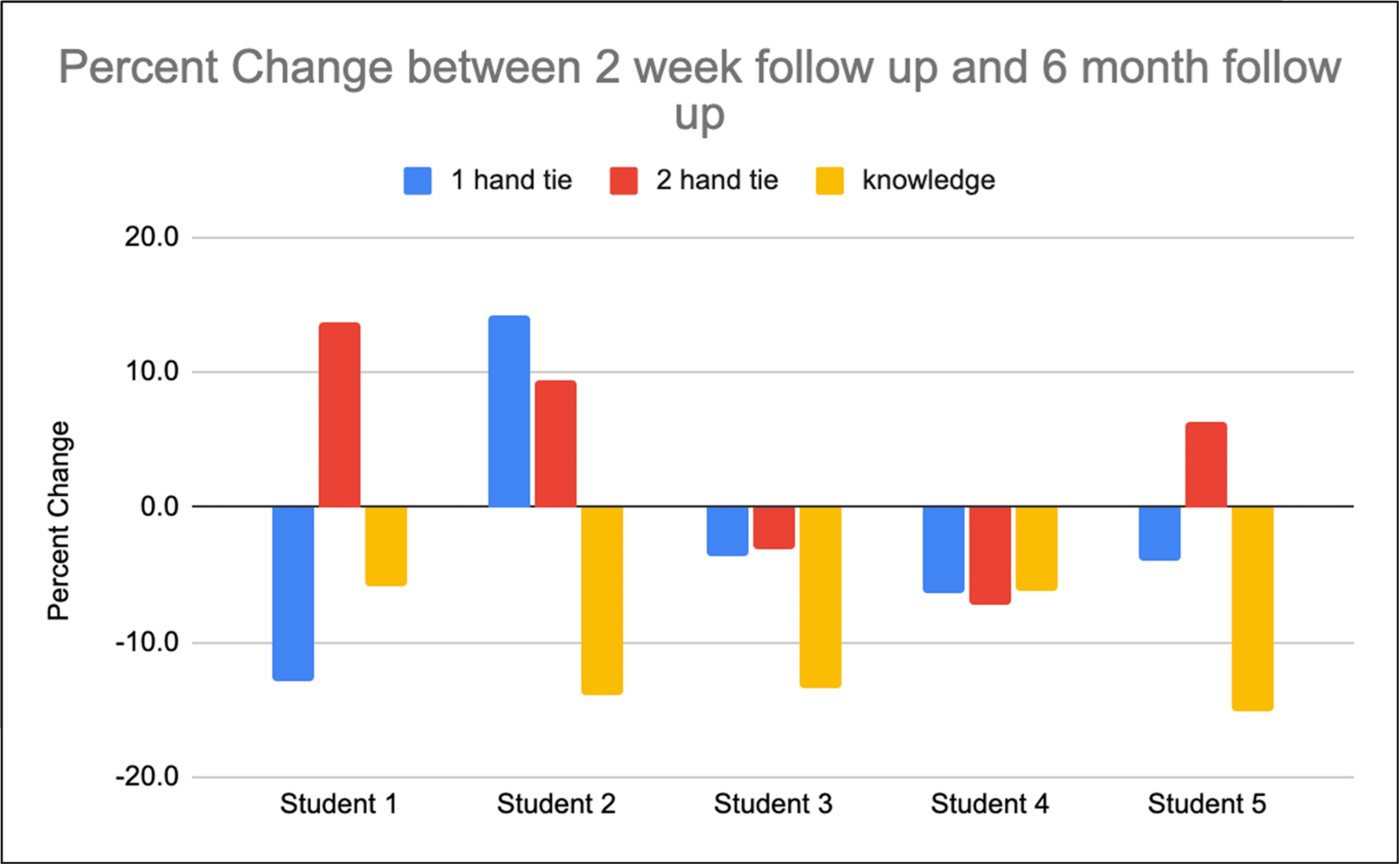
Difference between two-week and six-month follow-up in skills and knowledge

Individually, all students were still above their baseline in regard to knowledge, one-hand and two-hand knots at the six-month mark. (Figures 1-3). From their two-week follow-up to their six-month follow-up, four students regressed in their one-hand tie skills, two students regressed in their two-hand skills, and all students regressed in their knowledge (Figure 4).

The follow-up survey showed that students preferred interactive sessions, predominantly ones with interactions with residents and faculty, but also journal clubs and case conferences (Table 3). All students agreed that the rotation met their expectations and would be willing to pay approximately $100 for it. The questions regarding effective and ineffective sessions were left open-ended, and the results in Table 3 show what students listed in their responses. Fischer's exact test was used to analyze the significance between interactive and non-interactive sessions for efficacy, and interactive sessions were found to be more effective with a p-value of 0.003.

**Table 3:** Survey results after rotation

Questions	Number of responses
Did this rotation meet your expectations?	
Strongly agree	3
Agree	2
What would you pay for this rotation?	
$100-200	3
$50-100	2
What was effective about this rotation?	
Interactive sessions	14 (p=0.003)
Case conferences	3
Simulated patients	1
Journal clubs	1
Other interactions with residents	4
Other interactions with faculty	5
Non-interactive sessions	5
Landmark journals	4
Surgical videos	1
What was ineffective about this rotation?	
Interactive sessions	0
Non-interactive sessions	6
Other readings	2
Surgical simulation tour	3
Electronic medical record review	1

## Discussion

All students who participated in the rotation retained a statistically significant portion of their skills over six months. Although skill levels declined from the two-week to the six-month follow-up, the difference was not statistically significant. Studies analyzing medical trainees' skills and retention after training have shown that factors related to skills decay are directly associated with the time since training, the frequency with which those skills are performed, and the quality of the feedback they received [7,8]. In this study, all students were actively pursuing surgical residency and were likely to practice these hand-tie skills after the rotation. Additionally, hand-tie skills are simple to set up and replicate multiple times. Third, each student received specific one-on-one feedback after each training session.

As with skills, knowledge of a topic decays over time after initial teaching. A large systematic review of medical trainees in advanced cardiac life support found that knowledge decay occurs after reassessment at six months to one year [9]. Studies examining retention factors among medical trainees found that annual training improved retention of infrequent topics, such as obstetrical emergencies and basic life support [10,11]. As in this study, after six months, there was a statistically significant decrease in knowledge of vascular surgery between the two-week and six-month follow-ups. All students in this study indicated that they were pursuing a vascular surgery residency, but most also stated that this was their only available "away rotation." This would significantly affect exposure to vascular surgery experience and the repeat assessment of topics such as vascular anatomy, peripheral arterial disease management, vascular imaging, and vascular trauma over the next six months.

Virtual rotation feedback from other studies has shown that students prefer interactive sessions [12-14]. Students opted into virtual rotations to gain face time with attendings and residents ahead of the match, improving their chances of matching at that site. Additionally, students prefer interactive sessions for a more engaging learning experience, where they can ask questions and improve their competency in the topic at hand. All students from this rotation requested more interactive sessions with faculty and residents.

This study has several important limitations. The sample size is extremely small and limited to a single institution, which restricts generalizability. There was no control group for comparison; while the baseline, two-week, and six-month data served in part as a control, the subjects remained the same, with no other control to compare to. This was due to the short amount of time available to organize the rotation, as well as limitations in how many students could enroll due to its uniqueness at the time. Follow-up data on whether students had additional vascular exposure or independent study after the rotation were not collected. Not having a standardized process for the knowledge test, knot-tying assessment, and survey design limits reproducibility as well. However, this rotation had been created in a very short amount of time to meet the demands of medical education during the pandemic. As this was not a commonality for medical rotations, let alone surgically inclined rotations, creating a widespread standardized protocol and guideline across multiple centers was not feasible. Future studies addressing these shortcomings should include larger and more diverse cohorts, collect detailed follow-up exposure data, and provide comprehensive methodological descriptions to improve reproducibility and interpretability. Another way to reduce variability would be to create a standardized curriculum across various institutions, addressing learning objectives, skills tests, and other parameters expected from a vascular surgery rotation.

## Conclusions

This pilot study suggests that a virtual vascular surgery rotation may be associated with short-term improvements in knowledge and sustained improvements in knot-tying skills among a small group of fourth-year medical students. However, knowledge gains declined by six months, and the results should be interpreted as preliminary and hypothesis-generating due to the uncontrolled design and small sample size. Further research with larger cohorts, more rigorous methodology, and standardization of objectives, learning objectives, and milestones is needed to draw definitive conclusions about the effectiveness of virtual surgical rotations and strategies to enhance long-term knowledge retention.
